# A complex of cadherin 17 with desmocollin 1 and p120-catenin regulates colorectal cancer migration and invasion according to the cell phenotype

**DOI:** 10.1186/s13046-024-02956-6

**Published:** 2024-01-24

**Authors:** Rubén A. Bartolomé, Laura Pintado-Berninches, Ángela Martín-Regalado, Javier Robles, Tania Calvo-López, Marina Ortega-Zapero, Celia Llorente-Sáez, Issam Boukich, María Jesús Fernandez-Aceñero, J. Ignacio Casal

**Affiliations:** 1 Department of Biomolecular Medicine, Centro de Investigaciones Biológicas Margarita Salas (CSIC), Ramiro de Maeztu 9, Madrid, 28040 Spain; 2 Biochemistry Department, Universidad Autónoma de Madrid, Madrid, Spain; 3 Protein Alternatives SL. Tres Cantos, Madrid, Spain; 4 Pathology Service. Hospital Clínico San Carlos, Madrid, Spain; 5 Fundación de Investigación Biomédica del HCSC (FIBHCSC), Madrid, Spain; 6 Present address: Facultad de Veterinaria, Universidad Complutense de Madrid, Madrid, Spain

**Keywords:** DSC1, CDH17, p120-catenin, Metastasis, Colorectal cancer, Therapeutic peptide

## Abstract

**Background:**

Cadherin-17 (CDH17), a marker of differentiation in intestinal cells, binds and activates α2β1 integrin to promote cell adhesion and proliferation in colorectal cancer (CRC) metastasis. Furthermore, CDH17 associates with p120- and β-catenin in a manner yet to be fully elucidated. In this report, we explored the molecular mediators involved in this association, their contribution to CRC dissemination and potential therapeutic implications.

**Methods:**

Proteomic and confocal analyses were employed to identify and validate CDH17 interactors. Functional characterization involved the study of proliferation, migration, and invasion in cell lines representative of various phenotypes. Immunohistochemistry was conducted on CRC tissue microarrays (TMA). In vivo animal experiments were carried out for metastatic studies.

**Results:**

We found that desmocollin-1 (DSC1), a desmosomal cadherin, interacts with CDH17 via its extracellular domain. DSC1 depletion led to increased or decreased invasion in CRC cells displaying epithelial or mesenchymal phenotype, respectively, in a process mediated by the association with p120-catenin. Down-regulation of DSC1 resulted in an increased expression of p120-catenin isoform 1 in epithelial cells or a shift in cellular location in mesenchymal cells. Opposite results were observed after forced expression of CDH17. DSC1 is highly expressed in budding cells at the leading edge of the tumor and associates with poor prognosis in the stem-like, mesenchymal CRC subtypes, while correlates with a more favorable prognosis in the less-aggressive subtypes. In vivo experiments demonstrated that DSC1 silencing reduced tumor growth, liver homing, and metastasis in CRC mesenchymal cells. Furthermore, a synthetic peptide derived from CDH17, containing the NLV motif, effectively inhibited invasion and liver homing in vivo, opening up new possibilities for the development of novel therapies focused on desmosomal cadherins.

**Conclusions:**

These findings shed light on the multifaceted roles of CDH17, DSC1, and p120-catenin in CRC metastasis, offering insights into potential therapeutic interventions for targeting desmosomal cadherins in poorly-differentiated carcinomas.

**Supplementary Information:**

The online version contains supplementary material available at 10.1186/s13046-024-02956-6.

## Background

Metastasis continues to be the major cause of mortality in colorectal cancer (CRC). Alterations in cadherins, catenins and associated signaling pathways play a central role in cell–cell adhesion, cancer progression and metastasis [1, 2]. For cancer migration and invasion, intercellular junctions and their components need to be modified in a dynamic way. Four types of intercellular junctions are present in vertebrates: adherens junctions, tight junctions, desmosomes and gap junctions. Cadherins are major components of adherens junctions and demosomes. In normal cells, adherens junctions (AJs) and desmosomal cadherins link the actin and intermediate filament cytoskeletons, respectively, to the plasma membrane at sites of cell–cell adhesion [3]. Desmosomal cadherins (i.e. desmocollins and desmogleins) are expressed in a cell type- and differentiation-dependent manner [4]. They appeared late in evolution and were designed for resisting high mechanical stress, i.e. in the skin or the heart [2]. To note that normal colon epithelium only expresses desmocollin 2 (DSC2) [5]. However, a desmocollin switch from DSC2 to DSC1/3 has been reported in CRC [5], in a similar way to the classical cadherin switching, or replacement of E-cadherin by N-cadherin, that has been associated with epithelial-mesenchymal transition (EMT) in multiple cancers [6]. The EMT process is driven by cellular plasticity, which allows cancer epithelial cells to acquire the mesenchymal phenotype required for cancer progression and dissemination [7]. Still, the role of non-classical (i.e. cadherin 17 (CDH17)) and desmosomal cadherins in EMT and other cancer processes remains poorly understood [1]. Indeed, conflicting results propose a role for desmosomal cadherins either as suppressors or promoters of tumor metastasis (see [8] for a review).

CDH17 is an atypical 7-domain cadherin characterized by an amino-terminal duplication of the first two cadherin repeats [9], and the presence of a truncated cytoplasmic domain that lacks the two armadillo domains present in the classical type I cadherins [9]. CDH17 expression localizes to the basolateral domain of hepatocytes and enterocytes, and has been associated with lipid rafts [10]. The short cytoplasmic tail of CDH17, lacking catenin binding sites, facilitates a high lateral mobility for CDH17 in the plasma membrane [10]. Regulation of lateral mobility is important for controlling cellular adhesion [11]. However, initial reports of CDH17 location in normal rat intestinal cells did not indicate its presence in AJs or demosomes [12]. Human CDH17 is only expressed in normal adult intestine, but is re-expressed in stomach, pancreatic and hepatocellular carcinomas [13]. CDH17 expression is regulated by CDX2, an intestine-specific homeobox transcription factor, which plays a key role in intestinal differentiation and homeostasis [14]. CRC tumors show increased CDH17 expression at metastatic stage in well- and moderately-differentiated tumors [15]. Consequently, poorly-differentiated tumors lack CDX2 expression and, usually, show weak CDH17 expression [15, 16].

CDH17 promotes CRC liver metastasis using its RGD motif for binding α2β1 integrin [15, 17]. However, CDH17 role in progression and metastasis is likely to go beyond its association with α2β1 integrin for increased adhesion and proliferation [1, 15, 17]. Indeed, CDH17 was described to activate the Wnt/β-catenin signaling pathway in hepatocellular carcinoma [18]. Furthermore, we observed the association of CDH17 with p120- and β-catenin after proteomic analysis of KM12SM cells [15], which has been classified as consensus molecular subtype 1 (CMS1) cell line. Classical cadherins, like E- or N-cadherin, directly bind p120- and β-catenin through their cytoplasmic domains, but given the short cytoplasmic tail of CDH17, its association with p120- and β-catenin is likely mediated by other proteins [15]. The role of p120-catenin in cancer progression is highly complex and relies on the relative expression of the different p120-catenin isoforms [19]. Epithelial cells mainly express isoforms 3 and 4, whereas isoform 1 (full-length) is the most commonly expressed isoform in mesenchymal cells [19]. Isoform 1 associates with enhanced cell invasion in colon cancer cells, whereas epithelial isoforms 3 and 4 increase cell viability and proliferation [20]. In a similar way to the cadherin and desmocollin switch, there is also a p120-catenin switch from isoform 3 to isoform 1 associated with EMT and likely regulated by mediators like Snail [21]. To note that p120-catenin is essential for the stabilization of classical cadherins at the cell surface and vice versa [22]. Loss of E-cadherin expression results in p120-catenin relocation for promoting invasiveness through regulation of Rho GTPases [23], but no effects of p120-catenin have been described for atypical cadherins stability. Some desmosomal proteins (i.e. desmogleins (DSG)) also bind p120-catenin through the cytoplasmic domain [24, 25].

Considering the different effects reported for cadherins and catenins in cancer progression according to the cell phenotype, we explored the interactome of CDH17 in two different CRC cell lines, RKO and HT-29, representatives of undifferentiated CMS4 and colon-like CMS3 subtypes, respectively [26] in order to find novel associations of CDH17 in the different CRC subtypes. Subsequently, we assessed the functional effects of the identified proteins across various cell lines, including epithelial, colon-like, KM12SM, HT-29, and mesenchymal SW620 and HCT-116 cells. To note that mesenchymal SW620 and HCT-116 cell lines belong to the CMS4 subtype and are representative of poorly-differentiated tumors, whereas epithelial, colon-like, KM12SM was classified as CMS1, the immune/microsatellite instability subtype [26, 27]. Our investigation revealed an association between CDH17 and desmocollin 1 (DSC1), a desmosomal cadherin, in both cell lines. Functional analysis of the protein complex in different cell types indicated a dual role for DSC1 in regulating the invasive behavior of CRC cells according to the cell phenotype (epithelial/mesenchymal) and the cancer subtype. CDH17/DSC1 effects were mediated through the recruitment of p120-catenin, which regulates the actin polymerization required for the invasiveness of the cells. Furthermore, DSC1 exhibited significant expression in budding cells within human tumor samples, correlating with poor prognosis in CRC mesenchymal subtypes. Remarkably, CDH17/DSC1 protumorigenic effects were inhibited by a synthetic peptide encompassing the NLV binding motif. These results uncover promising avenues for the development of novel therapeutic strategies focused on the inhibition of CDH17/DSC1 interactions.

## Methods

### Cell culture conditions, transfections. peptides and antibodies

Human colorectal cancer cell lines HT-29, RKO and HCT-116 were purchased from the ATCC (USA), SW620 was from the ECACC (Wiltshire, UK) and KM12SM was obtained from Dr. I Fidler (MD Anderson Cancer Center, Houston, TX, USA). All cells were cultured in DMEM (Invitrogen, Carlsbad, CA, USA) containing 10% FCS (Invitrogen) and antibiotics at 37ºC, in a 5% CO_2_ humidified atmosphere and passaged less than 6 months for all the experiments. Preparation of pcDNA3.1 vectors containing wt CDH17 and CDH17-RAD was previously described [17]. CDH17-RAD mutant contains a mutation in the RGD motif of CDH17 that prevents the binding to and the activation of α2β1 integrin [17]. Cell transfection procedures and reagents are described in Additional Information.

The synthetic CDH17 peptide LNPAKNPSYNLVISVKDM was synthesized using solid phase chemistry with a Focus XC instrument (AAPPtec, Lousville, KY, USA). The antibodies against CDH17 were 243,391-AP (Proteintech, Rosemont, IL, USA) used in Western blot and immunofluorescence, and H-167 and H-1 (Santa Cruz Biotechnologies, Dallas, TX, USA) used in immunoprecipitation and immunohistochemistry, respectively. The antibodies anti-DSC1 were E-AB-13193 (Elabscience, Houston, TX, USA) for immunohistochemistry, and A-4 (Santa Cruz Biotechnologies) for Western blot and immunofluorescence. The antibodies FAK (D1), EGFR (1005) and RhoGDI (G-2) were from Santa Cruz Biotechnologies, whereas Src (AF648) antibody was from R&D Systems (Minneapolis, MN, USA). Anti-p120-catenin (610,134) for Western blot and anti-phospho-FAK (15,806,019) were from BD Biosciences (San Diego, CA, USA), and anti-α2 integrin (SN0752) from Invitrogen. The antibodies against p120-catenin (#59,854) for immunofluorescence, β-catenin (#8814), phospho-ERK1/2 (#9106), ERK (#4695), phospho-AKT (#3787), AKT (#2920), phospho-JNK (#9255), JNK (#9258) and phospho-Src (#2101) were purchased from Cell Signaling Technologies (Danvers, MA, USA). The antibodies against β-actin (TA811000S) and Lamin B (12,987–1-AP) were from Origene (Rockville, MD, USA) and Proteintech, respectively.

### Immunoprecipitation

KM12SM and SW620 cells were lysed in 1% Igepal (NP-40), 100 mM NaCl, 2 mM MgCl_2_, 10% Glycerol in 50 mM Tris–HCl with protease (Roche, Basel, Switzerland) and phosphatase inhibitors (Sigma-Aldrich, St. Louis, MO, USA). 1 mg of cell lysate was incubated with Protein G-sepharose beads (Sigma-Aldrich) and anti-CDH17 or control antibodies for 16 h. Immunoprecipitates were washed in lysis buffer, resuspended in Laemmli buffer, and resolved in SDS-PAGE, which were analyzed by Western blot analysis (described in Additional Information).

For proteomic analysis of CDH17-coimmunoprecipitated proteins, RKO and HT-29 cells previously transfected with vectors encoding for CDH17 were lysed and subjected to immunoprecipitation as before [15]. Co-immunoprecipitated proteins were in-gel digested with trypsin and peptides subjected to mass spectrometry analysis (Fig. S1A) (described in Additional Information). Proteins were considered positive for interaction when they showed at least twice more unique peptides than the control IP. Analysis of CDH17 co-immunoprecipitated proteins was performed with DAVID database (https://david.ncifcrf.gov/) using the Gene Ontology (GO) annotation GOTERM_BP_FAT.

### Confocal microscopy

Confocal microscopy methods are described in Additional Information.

### Cell adhesion, migration, invasion, proliferation and actin polymerization assays

Cell adhesion to Matrigel, invasion through Matrigel and MTT assays were performed as previously described [28]. Wound healing assays to assess cell migration were carried out as previously described [29]. For actin polymerization, transfected cells were detached with 2 mM EDTA, fixed with 7.4% formaldehyde, permeabilized with 1 mg/mL lysophosphatidylcholine (Sigma-Aldrich) and stained with Phalloidin-Alexa 546 (Invitrogen). Labeled cells were analyzed in a Cytomics FC500 flow cytometer (Beckman Coulter). Peptide NLV was used between 0.3–30 μg/mL in the different experiments.

### Immunohistochemistry

A tissue microarray (TMA) containing the invasive front of CRC primary tumors (*n* = 30) was prepared and obtained from the Surgical Pathology Department of the Hospital Clínico San Carlos (Madrid, Spain) as previously described [30]. Slides were deparaffinized for antigen retrieval using citrate sodium buffer (pH 6.0) for 25 min and incubated with anti-DSC1 or anti-CDH17 antibodies (1 μg/mL) overnight at 4ºC. Then, slides were incubated with a peroxidase-labelled polymer conjugated to goat anti-mouse and goat anti-rabbit antibodies (EnVision Dual Link system-HRP. DAKO. Denmark) for 30 min at room temperature and the reaction was developed using diaminobenzidine as chromogen, and hematoxylin for counterstaining. Images were acquired with a Leica DM2000 LED microscope (Leica, Wetzlar, Germany) using a × 20 objective and processed with the LAS-V4.8 Leica software.

### Metastasis experiments in nude mice

The ethics committees of the CSIC and Community of Madrid approved all protocols used (PROEX 140/18). Liver homing assessment was performed as previously described [28]. In brief, mice (*n* = 3) were inoculated in the spleen with control or DSC1-silenced KM12SM, SW620 and HCT-116 (10^6^ cells) or cells treated with the CDH17 NLV peptide (10 μg/mL). After 96 h, mRNA was isolated from liver using Trizol (Thermo Fisher Scientific, Waltham, MA, USA) and subjected to RT-PCR with M-MLV retrotranscriptase (Promega, Madison, WI, USA) and Taq DNA polymerase (Thermo Fisher Scientific) to amplify human GAPDH and, as control, murine β-actin. The detection of human GAPDH indicates liver colonization by CRC cells. For assessment of tumor growth, the same transfectants were inoculated subcutaneously (5 × 10^6^) in NSG mice (*n* = 6). After 6 days, subcutaneous tumors were removed and measured. For Kaplan–Meier survival curves, HCT-116 transfectants or control cells were inoculated in NSG mice (*n* = 6). 24 h after inoculation, mice were subjected to splenectomy to avoid local growth of tumors. Mice were monitored daily for signs of disease including reduced mobility, loss of appetite, tumor detection by palpation, self-isolation, dermatitis, etc. After relevant sign detection, mice were euthanized and subjected to necropsy to verify the presence of liver metastases.

### Prognostic value of DSC1 in colorectal cancer patients

In silico analysis of DSC1 prognostic value in CRC patients is described in Additional Information.

### Statistical analyses

Histograms showed the average value, indicating the standard deviation as error bars from three independent experiments. Data were analyzed by Student’s t test for two conditions or by one-way ANOVA followed by Tukey–Kramer multiple comparison test for more than two conditions. Overall survival in Kaplan–Meier survival analyses was estimated using log-rank test. In all analyses, the minimum acceptable level of significance was *P* < 0.05.

## Results

### CDH17 associates with desmosomal proteins in colorectal cancer cells

**Fig. 1:**
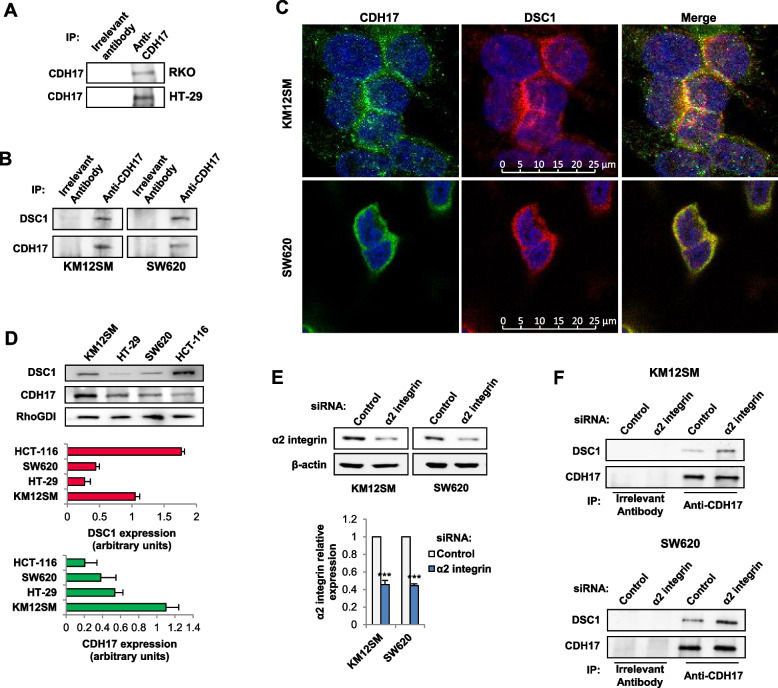
CDH17 associates with DSC1. **A** RKO and HT-29 cells were transfected with vectors encoding for CDH17. Transfectants were subjected to immunoprecipitation (IP) using anti-CDH17 or control antibodies, and the IPs were analyzed by Western blot to detect CDH17. **B** KM12SM and SW620 cells were lysed and the extracts subjected to IP as in **A**. IPs were analyzed by Western blot to confirm DSC1 and CDH17 co-immunoprecipitation. **C** Immunofluorescence showing the co-localization of CDH17 and DSC1 in KM12SM and SW620 cells. **D** Western blot analysis of the indicated CRC cell lines to detect DSC1 and CDH17. RhoGDI was used as a loading control. **E** The indicated cell lines were transfected with control or α2 integrin-targeting siRNAs. The α2 integrin expression levels were significantly inhibited by the siRNA transfection (***, *p* < 0.001). **F** Cell lysates from the transfectants were subjected to IP as in **A**. IPs were analyzed by Western blot to detect DSC1 and CDH17. Data are representative of three independent experiments

Full-length CDH17 was overexpressed in mesenchymal RKO and epithelial HT-29 CRC cells to explore CDH17 interactions in different cellular phenotype contexts and different CRC subtypes. To note that RKO cells exhibited negligible levels of CDH17 expression before transfection [15, 17]. Then, the transfectants were subjected to immunoprecipitation (IP) with anti-CDH17 and compared with a control antibody IP (Fig. 1A**)**, followed by mass spectrometry analysis (Table S1). CDH17 immunoprecipitated proteins obtained from mesenchymal RKO cells were fewer than those from HT-29 cells, which were more similar in number and function to those previously described for KM12SM [15] (Table S2, Fig. S1B, C). Indeed, the CDH17 IP proteins in HT-29 exhibited a 23% overlap with those from KM12SM [15], with 11% of the proteins sharing protein family similarities, particularly those associated with cell surface receptors and integrin-mediated signaling (Fig. S1C, D). The limited overlap in common proteins between RKO indicates significant differences in the CDH17 interactome network between the two cell lines. In HT-29, CDH17 showed associations with the regulation of actin filaments (ACTN1, SPTAN1, IQGAP1, CDC42, PFN1, ARPC2), and microtubules (TUBA1C, TUBB5, TUBB2C, MYH9), implying a role in cytoskeleton dynamics (Fig. S1C). Additionally, CDH17 was linked to the regulation of cell adhesion and integrin signaling (TLN1, RCC2, MYH9, CDC42). Another significant difference was the association of CDH17 with signaling pathways in epithelial cells, which was absent in RKO (Fig. S1D). Overall, these results support a larger implication of CDH17 in cytoskeleton dynamics and signaling pathways in epithelial cells than in CDH17-negative RKO cells.

Among the main interactors of CDH17 in both cell lines were DSC1 and junction plakoglobin (JUP), two desmosomal proteins (Table S1). Given that JUP is an intracellular protein binding to the cytoplasmic tail of desmocollins, including DSC1, we postulated that DSC1 might work as the direct partner of CDH17. Although this connection was not previously observed in KM12SM cells [15], the association of CDH17 with DSC1 drew our attention, as it suggests a potential novel mediator for CDH17-mediated functions that may explain the observed connections between CDH17 and the cytoskeleton dynamics. Considering the short length of the cytoplasmic region of CDH17, the interaction between CDH17 and DSC1 is likely to occur through their extracellular domains. To confirm the CDH17/DSC1 association, we conducted CDH17 IP experiments in KM12SM and SW620 cells, followed by western blot analysis of DSC1 (Fig. 1B). Furthermore, confocal microscopy studies revealed a significant co-localization of both proteins in the plasma membrane of KM12SM and SW620 cells (Fig. 1C). Regarding expression in colon cancer cell lines, KM12SM and HCT116 exhibited high levels of DSC1, while CDH17 was more abundant in the epithelial cell lines KM12SM and HT-29 (Fig. 1D). Finally, we explored the association of the α2β1 integrin with the DSC1/CDH17 complex. After silencing the α2 integrin subunit in KM12SM and SW620 cells (Fig. 1E), we observed an increase in the association of DSC1 with CDH17 following CDH17 IP (Fig. 1F). This observation suggests a potential competition between these molecules for binding to CDH17.

### The CDH17/DSC1 complex promotes a different regulation of the cell adhesion, migration and invasion according to the colorectal cancer subtype

**Fig. 2:**
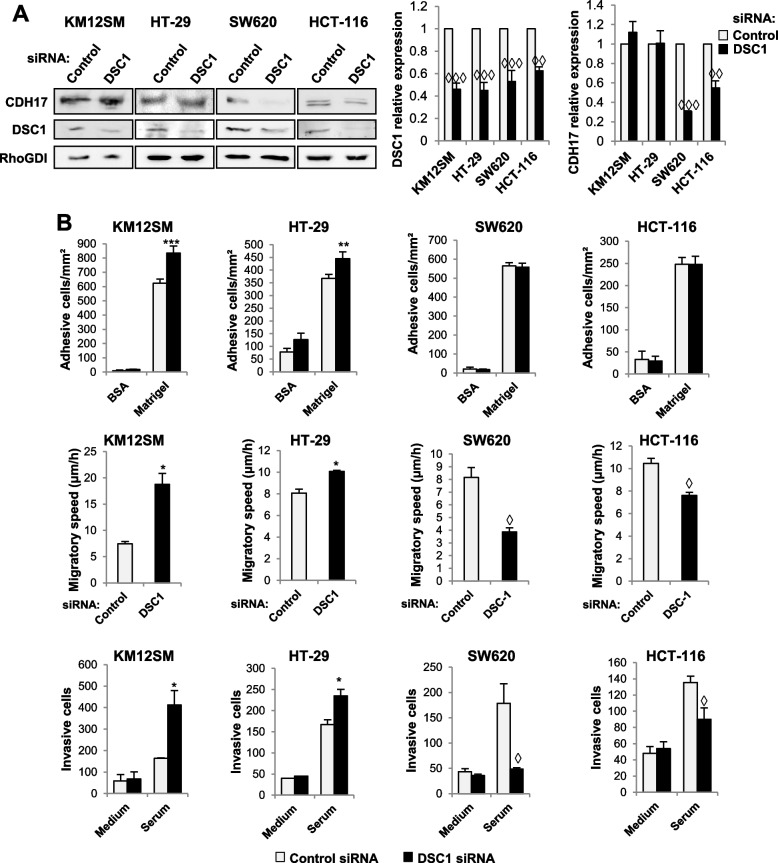
DSC1 regulates cell adhesion, migration and invasion. **A** The indicated cell lines were transfected with control or DSC1-targeting siRNAs, and the extracts analyzed by Western blot to detect DSC1, CDH17 and, as a loading control, RhoGDI. DSC1 or CDH17 expression levels were significantly inhibited by the siRNA transfection (◊◊, *p* < 0.01; ◊◊◊, *p* < 0.001). **B** The same transfectants were subjected to cell adhesion, wound healing and invasion. Cell adhesion, migration or invasion were significantly decreased (◊, *p* < 0.05) or increased (*, *p* < 0.05; **, p < 0.01; ***, *p* < 0.001) after DSC1 silencing. Data are representative of three independent experiments

**Fig. 3:**
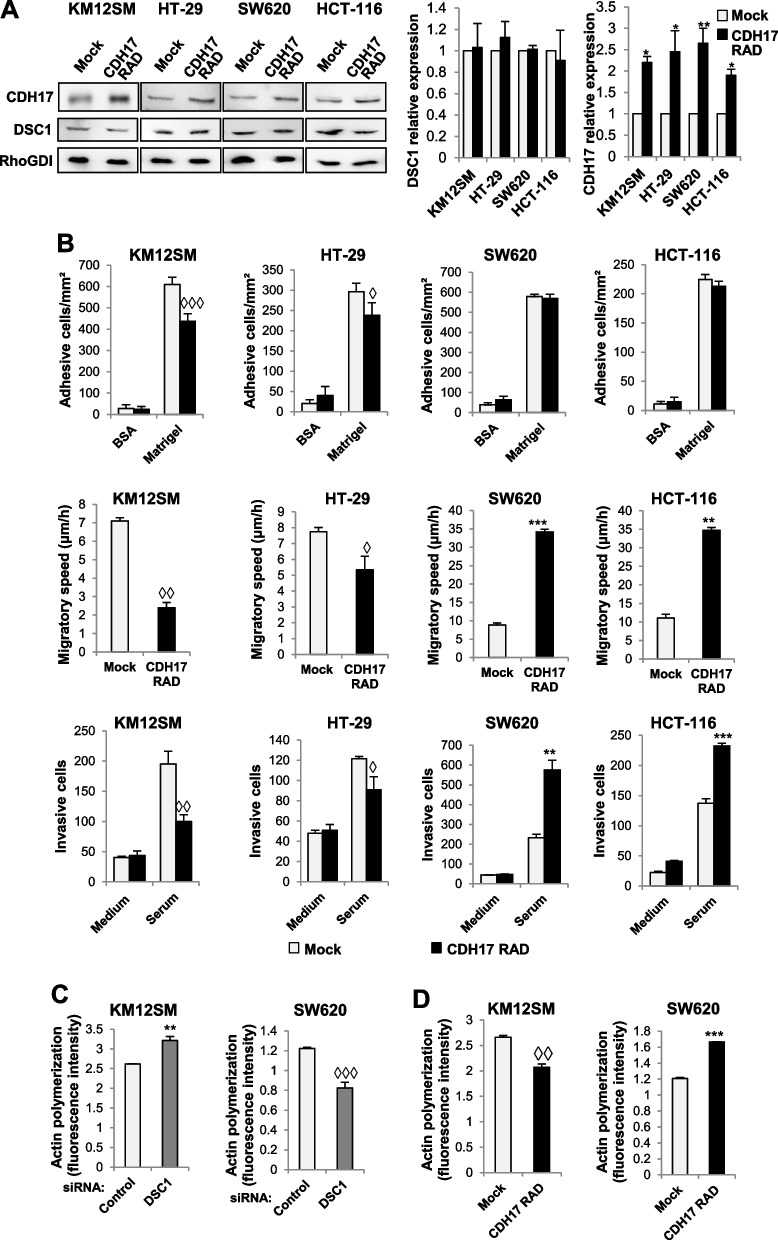
CDH17 overexpression regulates cell adhesion, migration and invasion in colon cancer cells. **A** The indicated cancer cell lines were transfected with empty vectors (Mock) or vectors encoding for the mutant CDH17 RAD. Cell extracts were analyzed by Western blot to detect CDH17, DSC1 and, as loading control, RhoGDI. CDH17 expression levels were significantly increased by the transfection of CDH17 RAD vectors (*, *p* < 0.01; **, *p* < 0.01). **B** The same transfectants were subjected to cell adhesion, wound healing and invasion. **C** The control or DSC1-silenced transfectants were subjected to actin polymerization assays. **D** The same transfectants as in A were subjected to actin polymerization assays. Cell adhesion, migration, invasion or F-actin amount were significantly reduced (◊, *p* < 0.05; ◊◊, *p* < 0.01; ◊◊◊, *p* < 0.001) or enhanced (**, *p* < 0.01; ***, *p* < 0.001) after overexpression of CDH17 RAD or DSC1 silencing. Data are representative of three independent experiments

Then, the functional effects of the CDH17/DSC1 complex were explored in four representative cell lines. KM12SM and HT29 belong to the epithelial CMS1 and CMS3 subtypes, respectively, while undifferentiated SW620 and HCT116 have been assigned to the stem-like, mesenchymal CMS4 subtype. First, we investigated the effects of DSC1 depletion using two different siRNAs on the expression of CDH17 (Fig. 2A, Fig. S2A). We observed that following DSC1 silencing, CDH17 expression remained similar or exhibited a slight increase in epithelial cells, whereas in mesenchymal cells, a significant reduction in CDH17 expression was evident. Accordingly, cell adhesion, migration and invasion increased in epithelial KM12SM and HT-29; while cell migration and invasion were strongly reduced in mesenchymal SW620 and HCT-116 cells with both siRNAs (Fig. 2B, Fig. S2B). Second, we expressed the mutant CDH17 RAD to investigate whether the observed effects were integrin- or DSC1-dependent. To note that CDH17 RAD, while retaining its ability to bind DSC1, does not activate α2β1 integrin. Although no effects on DSC1 expression were observed (Fig. 3A), CDH17 RAD epithelial transfectants showed a decrease in cell adhesion, migration and invasion. Conversely, CDH17 RAD expression promoted migration and invasion in mesenchymal cells, suggesting an increased complex formation with DSC1 (Fig. 3B, 1F). Next, we investigated the potential of DSC1 to modulate cell signaling. Silencing DSC1 revealed a reduced activation of JNK and Src expression in both epithelial and mesenchymal cell lines (Fig. S3). In contrast, the induction of p-AKT was enhanced in SW620 cells following DSC1 silencing, while it remained unaffected in KM12SM cells. Neither pERK nor pFAK showed any alterations after DSC1 knockdown.

A key requisite for cell migration and invasion is the formation of lamellipodia, filopodia or invadosomas that are promoted by actin polymerization. Therefore, we assessed actin polymerization in DSC1 knocked-down and CDH17 RAD-expressing cells. Consistent with previous results, DSC1 silencing caused an increase on F-actin content in KM12SM cells and a decrease in SW620 cells (Fig. 3C). On the contrary, CDH17 RAD overexpression showed a decrease on actin polymerization in KM12SM and an increase in SW620 (Fig. 3D). These differences in actin polymerization, mediated by DSC1, likely explain the observed differences in the migration and invasion of the cells.

### DSC1 associates with p120-catenin

**Fig. 4:**
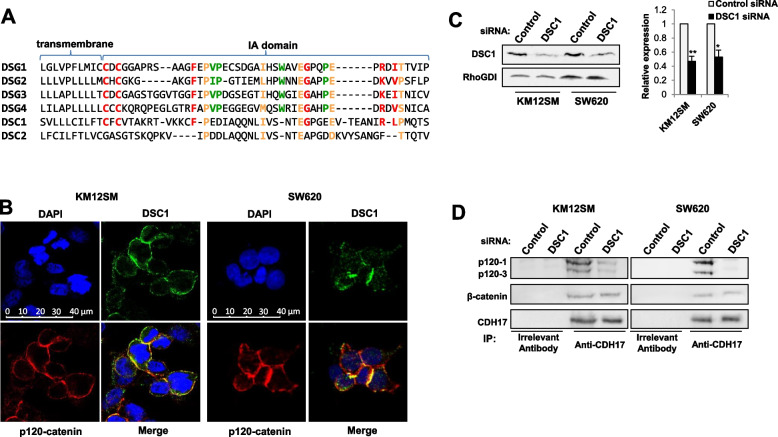
DSC1 associates with p120-catenin. **A** Alignment of IA domain of desmogleins and the homologous region of DSC1 and DSC2. Orange letters indicate common or similar residues in desmogleins and desmocollins, red ones in desmogleins and DSC1, and green ones those exclusive of desmogleins. **B** Confocal microscopy showing the colocalization of DSC1 and p120-catenin in KM12SM and SW620 cells. **C** The indicated cell lines were transfected with control or DSC1 siRNAs and the extracts were analyzed by Western blot to assess the knock down of DSC1. RhoGDI was used as loading control. DSC1 expression levels were significantly inhibited by the siRNA transfection (*, *p* < 0.05; **, *p* < 0.01). **D** The same transfectants were subjected to immunoprecipitation assays using control or anti-CDH17 antibodies, and the immunoprecipitates analyzed by Western blot to detect p120-catenin, β-catenin and CDH17. The immunoprecipitates were analyzed by Western blot to detect β-catenin and CDH17. **B**-**D** Data are representative of three independent experiments

Given that actin polymerization is modulated by p120-catenin mediated regulation of the Rho-GTPases, RAC1 and CDC42 [31], we explored the potential association between p120-catenin and DSC1 to mediate these effects. An in silico study revealed a significant homology between the cytoplasmic sequence of DSC1 and key residues in DSG2 or DSG3, which are known to associate with p120-catenin through the IA domain [25] (Fig. 4A). Interestingly, DSC1 showed higher homology than DSC2 with DSG2/3 (Fig. 4A). For a direct demonstration of the potential interaction, we carried out a confocal analysis of DSC1 and p120-catenin expression in KM12SM and SW60 cells. Results indicate an overlap staining between both proteins in the cell membrane for both cell lines, supporting the co-localization (Fig. 4B). Next, to verify whether DSC1 was the mediator of the association of p120-catenin with CDH17, knocked-down DSC1 transfectants in KM12SM and SW620 (Fig. 4C**)** were subjected to CDH17 IP. DSC1 silencing significantly inhibited the association of CDH17 with p120-catenin in both cell lines (and the loss of the triple complex) **(**Fig. 4D). In contrast, no alterations were observed for β-catenin. Together, these findings demonstrate that DSC1 associates with p120-catenin in CRC cells to build a triple functional complex CDH17/DSC1/p120-catenin.

### CDH17/DSC1 regulate p120-catenin isoform expression and cellular distribution

**Fig. 5:**
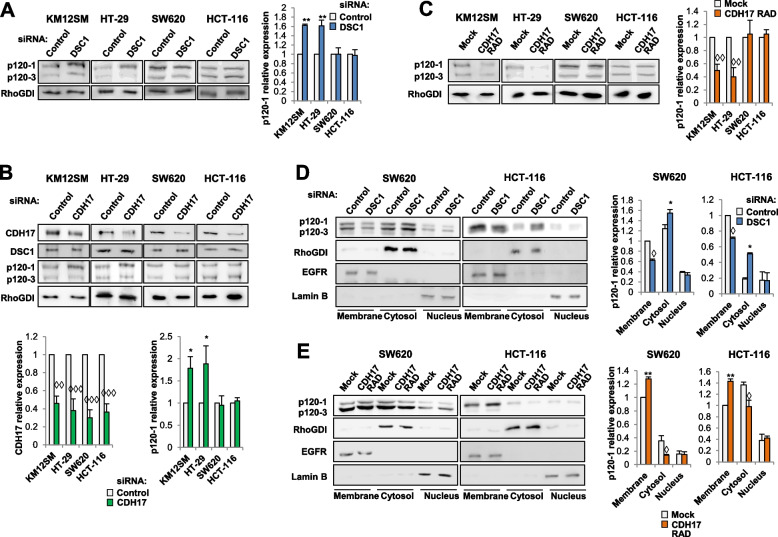
CDH17 and DSC1 regulate p120-catenin isoform 1 expression. **A**, **B** The indicated cell lines were transfected with control, DSC1 or CDH17 targeting siRNAs. Cell extracts were analyzed by Western blot to detect the indicated proteins. The indicated protein expression levels were significantly increased (*, *p* < 0.05; **, *p* < 0.01) or decreased (◊◊, *p* < 0.01; ◊◊◊, *p* < 0.001) after siRNA transfection. **C** The same cell lines were transfected with empty vectors (Mock) or vectors encoding for CDH17 RAD, and the cell extracts analyzed as before. **D**, **E** The indicated transfectants were subjected to subcellular fractionation and analyzed by Western blot. RhoGDI, EGFR and Lamin B were used as loading controls for cytosolic, membrane and nuclear fractions, respectively. The expression levels of p120-catenin isoform 1 in each subcellular fraction were significantly increased (*, *p* < 0.05; **, *p* < 0.01) or decreased (◊, *p* < 0.05) after DSC1 silencing or CDH17 RAD overexpression. Data are representative of three independent experiments

As p120-catenin isoforms 1 and 3 play opposite roles in tumorigenesis, we evaluated whether the CDH17/DSC1 complex could modulate the expression of these two isoforms in a different way. Thus, we determined the expression of p120-catenin isoforms 1 and 3 after DSC1 (Fig. 5A**)** or CDH17 (Fig. 5B) depletion, or CDH17 RAD transfection (Fig. 5C). DSC1 or CDH17 silencing caused an increase of p120-catenin isoform 1 expression in epithelial cells (⁓1.6-fold), without alterations in the mesenchymal cell lines (Fig. 5A, B). In contrast, CDH17 RAD transfectants promoted a reduced expression of p120-catenin isoform 1 in epithelial cells, without effect in mesenchymal cells (Fig. 5C). No major alterations were observed for p120-catenin isoform 3 expression in any cell line or condition. Since mesenchymal cells showed no alterations at the expression level of p120-catenin isoform 1, we hypothesized that the different effects on actin polymerization might be explained by a potential cellular relocation of p120-catenin isoforms in the mesenchymal cells. So, we carried out a cellular fractionation in SW620 and HCT-116 cell lines after DSC1 silencing or CDH17 transfection to test this hypothesis. DSC1 silencing decreased p120-catenin isoform1 expression in membrane, but increased cytosolic expression (Fig. 5D). Conversely, CDH17 RAD expression retained p120-catenin isoform 1 location into the cell membrane and decreased it in cytosol **(**Fig. 5E). These cellular alterations, while not resulting in overall changes in expression, indicate that CDH17/DSC1 expression differentially regulates the cellular location of p120-catenin isoform 1 in mesenchymal cells. So, whereas membrane p120-catenin isoform 1 presence favors migration and invasion, the cytosolic relocation reduces both effects in mesenchymal cells.

### DSC1 is present in budding cells and associates with poor prognosis in mesenchymal subtypes

**Fig. 6:**
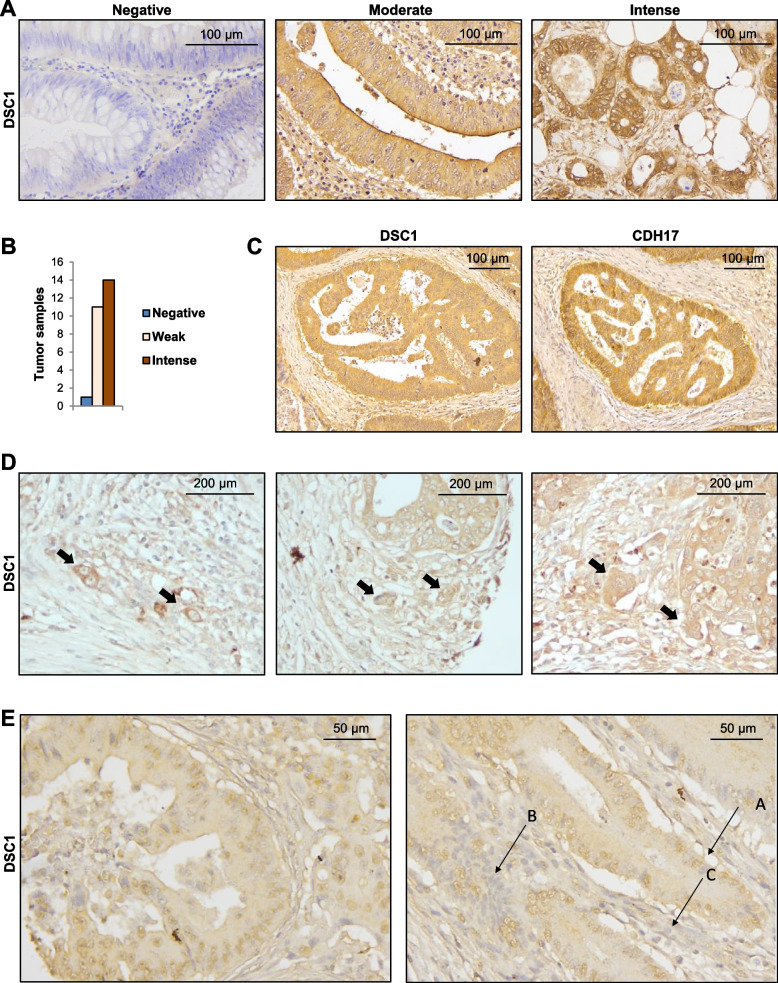
DSC1 expression in colon cancer samples. **A** Representative images of negative, moderate and intense DSC1 staining in colon cancer tissue samples subjected to immunohistochemistry. **B** Quantification of DSC1 staining in the analyzed tumor samples. **C** Representative images of DSC1 and CDH17 staining in serial tissue sections of colon cancer samples. **D** Representative images of the tumor invasive front showing the expression of DSC1 in budding cells (black arrows). **E** Representative images of DSC1 nuclear staining. Cells labeled with A and B showed positive and negative nuclear staining of tumor cells, respectively, whereas the C region indicates negative nuclear staining in fibroblasts

To study the clinical value of DSC1 expression and its potential association with the invasive front of the tumor, we performed IHC analysis of DSC1 in a tissue microarray containing biopsies of CRC primary tumors that included the leading front (Fig. 6A**)**. We noticed strong membrane staining in about 54% of the tumors, whereas moderate staining was observed in 42% and negative in 4% of the samples (Fig. 6B). Additionally, a parallel staining of DSC1 and CDH17 in serial sections revealed a significant correlation between DSC1 and CDH17 expression at the tumor (Fig. 6C). Interestingly, we observed DSC1 expression in budding cells located at the tumor leading front, enclosed within the stroma compartment (Fig. 6D). Moreover, a significant nuclear staining of DSC1 was observed in some tumors (Fig. 6E), suggesting a translocation of DSC1 to the nucleus, similar to that described for E-cadherin [32]. It is noteworthy that nuclear expression of DSC1 was detected in some sections of the tumors, whereas other sections remained free of nuclear expression, as observed in other components of the microenvironment like fibroblasts (Fig. 6E). To further assess the clinical significance of DSC1, we analyzed the correlation between mRNA expression levels and poor prognosis across various datasets, using two distinct CRC classifications, CMS and CRIS [27, 33]. In both classifications, high expression of DSC1 was associated with unfavorable outcome in the highly aggressive mesenchymal subtypes, CMS4 and CRIS-B, supporting a correlation between high expression of DSC1 and the mesenchymal, invasive CRC subtypes (Fig. S4). In contrast, DSC1 expression was associated with good prognosis in less aggressive subtypes, such as CMS2, CRIS-C or CRIS-E.

### DSC1 regulates liver homing and metastasis in mesenchymal colon cancer cells

**Fig. 7:**
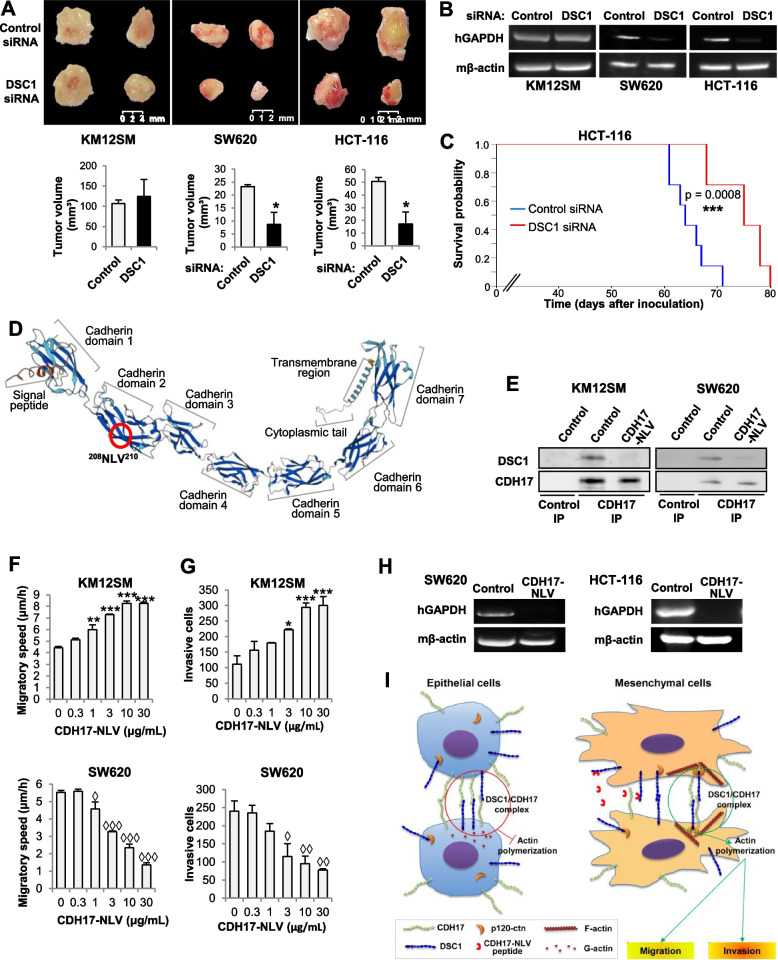
DSC1 promotes tumor growth and liver metastasis of colon cancer cells. **A** The indicated cell lines were transfected with control or DSC1-targeting siRNAs. The transfectants were inoculated subcutaneously. After 6 days, tumors were retrieved and measured. Tumor growth was significantly inhibited after DSC1 knock down (*, *p* < 0.05). **B** The same transfectants were inoculated in the spleen of nude mice, which were euthanized after 96 h. RNA from livers were isolated and subjected to RT-PCR to detect human GAPDH. As control, mouse β-actin was also amplified. **C** Kaplan–Meier survival of mice inoculated in the spleen with control or DSC1-silenced HCT-116 cells. Survival time was significantly enhanced in mice inoculated with DSC1-silenced cells (***, *p* < 0.001). **D** CDH17 structure showing the location of the NLV motif. **E** The indicated cell lysates were subjected to immunoprecipitation using control or anti-CDH17 antibodies in the presence or absence of the CDH17-NLV peptide (10 μg/mL). Immunoprecipitates were analyzed by Western blot to detect DSC1 and CDH17. **F**, **G** The indicated cell lines were subjected to migration **F** or invasion **G** assays in presence of increasing concentrations of the CDH17-NLV peptide. Migratory speed or number or invasive cells were significantly increased (**, *p* < 0.05; ***, *p* < 0.001) or reduced (◊, *p* < 0.05; ◊◊◊, *p* < 0.001) after treatment with the peptide. Data are representative of three independent experiments. **H** SW620 and HCT-116 were exposed to the CDH17-NLV peptide (10 μg/mL) and inoculated in the spleen of nude mice. Liver colonization was assessed as in E. **I** Functional model of the CDH17/DSC1 complex in colon cancer cells. In mesenchymal cells, the CDH17/DSC1 complex recruits p120-1 into the cell membrane, promoting actin polymerization, cell migration, invasion, and leading to an increase of the metastatic capacity of the colon cancer cells

Considering the localization of DSC1 in CRC budding cells, we proceeded to investigate the in vivo consequences of targeting DSC1 using mouse models for tumor growth, liver homing and metastasis. First, KM12SM, SW620 and HCT-116 cells, knocked down for DSC1 with a specific esiRNA, were subcutaneously injected in the flanks of Swiss nude mice and allowed to grow for 6 days. Then, the mice were sacrificed, and the tumors were surgically removed and assessed for their volume. DSC1 silencing significantly delayed the growth of mesenchymal cells-derived tumors (SW620 and HCT-116), but did not affect the growth of epithelial KM12SM xenografts (Fig. 7A). Next, KM12SM, SW620 and HCT-116 cells transfected with DSC1-targeting or control esiRNAs were tested for liver homing capacity after inoculation in the spleen of Swiss nude mice. After 96 h, RNA was isolated from the livers and subjected to RT-PCR for human GAPDH amplification as a surrogate. In KM12SM cells, DSC1 silencing did not affect the ability to colonize the liver, likely because CDH17 expression levels were not affected. In contrast, SW620 and HCT-116 cells homing was severely impaired after DSC1 depletion, as human GAPDH was barely detected (Fig. 7B). Next, we investigated the effects of DSC1 silencing on liver metastasis progression and survival. After inoculating metastatic mesenchymal HCT-116 KD and scrambled cells in the spleen of NSG mice, a Kaplan–Meier analysis indicated a significant increase in survival of mice treated with the silenced HCT-116 cells (*p* value = 0.0008) (Fig. 7C). SW620 cells were not tested because their low metastatic capacity. Collectively, these results indicate that DSC1 targeting inhibits tumor growth, homing and liver metastasis of CRC mesenchymal cells.

### Targeting CDH17/DSC1 complex with a NLV-containing synthetic peptide inhibits metastatic progression in colon cancer cells

Although there were no previous reports of binding between cadherins and desmocollins, E-cadherin was reported to bind DSG2 in the nascent desmosomes using the residue Leu175, within the motif 174-NLV-176 [24]. Interestingly, CDH17 contains the same DSG2-binding sequence in residues 207-NLV-210. The NLV motif is located in the second outermost extracellular domain of CDH17 (Fig. 7D). Structure prediction indicated a significant accessibility of the motif (Fig. S5). Therefore, we hypothesized that this motif might also be utilized by CDH17 for binding to DSC1. To test this hypothesis and examine the therapeutic potential of the DSC1/CDH17 interaction, we synthesized a CDH17 peptide: LNPAKNPSYNLVISVKDM. The selectivity of the NLV peptide for blocking the CDH17/DSC1 association was confirmed by CDH17 IP and western blot analysis in KM12SM and SW620 cells (Fig. 7E). Then, increased concentrations of the peptide NLV, starting at 1 µg/ml, inhibited the migratory and invasive capacity of mesenchymal SW620 cells, mirroring the silencing of DSC1, but increased the same capacities in KM12SM cells (Fig. 7F, G). Furthermore, we tested the capacity of the peptide to inhibit liver homing in DSC1-silenced mesenchymal cells. In both cell lines, SW620 and HCT-116, pretreatment with 10 µg/mL of the peptide abrogated the liver colonization capacity (Fig. 7H). Together, these observations support the therapeutic potential of disrupting the binding between CDH17 and DSC1 using a synthetic NLV peptide in mesenchymal, poorly-differentiated CRC cells.

## Discussion

In normal intestinal cells, DSC2 is preferentially expressed together with CDH17. However, during the neoplastic progression, a desmocollin switch occurs, causing the replacement of DSC2 by DSC1. In this study, we unveiled a dual role for DSC1, modulated by its interactions with both CDH17 and p120-catenin, in regulating the migration and invasion of CRC cells, according to the epithelial or mesenchymal characteristics. Furthermore, targeting DSC1 in the budding cells of the tumors by blocking its binding to CDH17 may represent a promising novel therapeutic strategy. Our conclusions were obtained from the following findings: 1) The immunoprecipitation of DSC1 with CDH17 in various cell lines, 2) The role of the CDH17/DSC1 complex in promoting migration and invasion in mesenchymal cells while inhibiting these capabilities in epithelial cells, 3) The regulation of p120-catenin expression in epithelial cells or its cytosolic/membrane translocation in mesenchymal cells, 4) The expression of DSC1 in tumor budding cells, together with an unfavorable prognosis for DSC1 expression in aggressive, mesenchymal CRC subtypes, and 5) The diminished migration, invasion, and liver homing of mesenchymal cells following DSC1 silencing or DSC1/CDH17 complex targeting with a synthetic NLV peptide. According to these observations, we have built a potential working model (Fig. 7I), where the CDH17/DSC1 complex promotes actin polymerization followed by migration and invasion by increasing p120-catenin isoform 1 membrane location in mesenchymal cells and the opposite effects in epithelial cells due to a lower p120-catenin-1 expression. Notably, these effects appear to be counteracted by a synthetic NLV peptide.

According to these and previous results, CDH17 might participate in, at least, two targetable complexes on the cell surface of CRC cells, either with α2β1 integrin or with DSC1/p120-catenin. However, the formation of these complexes might not occur simultaneously and will likely depend on the phenotype and differentiation status of the cell. In both cases, CDH17 uses its extracellular domain for the interactions with the integrin and the desmocollin. The formation of these complexes supports the notion of high lateral mobility for CDH17 within the cell membrane, which correlates with the presence of CDH17 in lipid rafts [1, 10]. It is noteworthy that also desmosomes preferentially partition into membrane lipid rafts [2]. We should remind that until now, no precise membrane localization has been assigned to CDH17, except for its typical basolateral localization in normal intestinal cells. Furthermore, it cannot be ruled out that other alterations in the membrane localization of CDH17 may occur during the neoplastic process, due to the cellular plasticity and the different tissue architecture of the tumor.

DSC1 knocked-out transgenic mice show epidermal blistering and abnormal differentiation, which indicates a critical relevance of DSC1 in the maintenance of strong intercellular adhesions and epidermal differentiation [34]. The adhesive characteristic of DSC1, together with its presence in CRC budding cells at the tumor front, might be relevant for the establishment of circulating tumor cell (CTC) clusters, a pivotal factor in the metastatic spread [35]. CTC clusters are known for their robust cell–cell contacts [36], facilitated by JUP-dependent intercellular adhesions [35]. To note that JUP was also immunoprecipitated with the CDH17/DSC1 complex, and was overexpressed in metastatic KM12SM cells compared to their non-metastatic counterparts [37]. Consequently, the presence of JUP in the CDH17/DSC1 complex should contribute to the stability and resistance to stress of CTC clusters, underscoring the significance of desmosomal proteins in CRC invasion and metastasis. It's also noteworthy that JUP (also known as γ-catenin) connects both, desmosomal and classical cadherins, playing an essential role in the assembly and interplay of AJs and desmosomes [3, 38]. Therefore, CDH17 might participate in the assembly of AJs and desmosomes through its interaction with DSC1 and JUP, playing a similar function to E-cadherin in the formation of nascent desmosomes [24].

In CRC, DSC2, which does not associate with p120-catenin [39], was reported to switch to DSC1 [5]. Here, we have demonstrated the capacity of DSC1 to associate with p120-catenin and CDH17 in metastatic CRC cells. Therefore, the DSC2/DSC1 transition emerges as a crucial requisite for the progression and metastasis of CRC. Overall, either the loss of DSC1 or the forced expression of CDH17 caused opposite effects in the expression (epithelial) or membrane location (mesenchymal) of p120-catenin to regulate cell migration and invasion, suggesting a similar mechanism of action for both molecules. Indeed, when both molecules were knocked down the effects were identical. Membrane-bound p120-catenin activates Rho-GTPases, enabling the formation of pro-migratory and pro-invasive structures such as lamellipodia or podosomes [40]. We hypothesized that the relocation of p120-catenin from the membrane to the cytosol might facilitate the internalization and degradation of CDH17, which might explain the loss of CDH17 in mesenchymal cells after DSC1 silencing. In contrast, the silencing of CDH17 has no discernible effect on the expression of DSC1.

In terms of clinical relevance, IHC analysis of the leading front of tumor samples revealed a high expression of DSC1 in CRC, particularly in migrating clusters formed by 2–3 budding cells. In some tumors, a clear staining of DSC1 was observed in the cell nucleus that indicates an unexpected nuclear translocation. These observations suggest that the CDH17/DSC1 complex may constitute a critical component for the formation and stability of CTCs in CRC. This hypothesis helps to explain the correlation of elevated expression of DSC1 with adverse outcomes in mesenchymal CMS4 and CRIS-B patients, representing the most aggressive subtypes of CRC [41]. The association of DSC1 with an adverse outcome was confirmed in mouse metastasis experiments. Indeed, silencing DSC1 in metastatic HCT-116 cells led to improved survival in treated mice.

Building on our previous findings that showed the efficacy of blocking CDH17-integrin interactions for inhibiting metastasis [28], our current results reveal novel therapeutic implications for CDH17 and underscore the value of blocking DSC1 association with CDH17 in the prevention of CTC clusters formation. Our group recently reported successful blocking of IL13 binding to IL13Rα2 using a synthetic peptide to prevent invasion and metastasis in CRC [42]. Here, we propose a similar approach, making use of the synthetic CDH17 peptide LNPAKNPSYNLVISVKDM, which was designed based on the homology with the E-cadherin sequence responsible for the binding to the desmosomal cadherin DSG2 [24]. Our results have demonstrated that this peptide was highly effective in hindering the migration, invasion, and homing capabilities of mesenchymal metastatic cells in vivo. Intriguingly, the motif NLV is also present in the protocadherin FAT4, which regulates EMT and the PI3K-AKT signaling pathway in CRC [43].

## Conclusions

Our proteomic strategy has revealed that the interaction between CDH17 and DSC1 promotes a dual effect on migration and invasion according to the cell phenotype context in CRC. These effects are mainly driven through the regulation of p120-catenin expression and cellular location. The p120-catenin plays an essential role for triggering actin polymerization that regulates the migration and invasion capacities of cancer cells. The clinical relevance of DSC1 in patients is underscored by its expression in budding clusters at the leading edge of the tumor. The cumulative results from the in vivo experiments emphasize the significance of DSC1 in driving the progression and metastasis in mesenchymal, poorly-differentiated, CRC subtypes. These pro-metastatic characteristics of DSC1 can be effectively inhibited using a specific NLV peptide derived from the CDH17 sequence. Nevertheless, our findings indicate that desmocollin effects are highly cancer cell context-dependent. In summary, blocking the formation of the CDH17/DSC1 complex offers an alternative therapeutic strategy for poorly-differentiated tumors, potentially complementing the inhibition of CDH17/α2β1 integrin binding in well- and moderately-differentiated tumors [28], and highlighting the relevance of CDH17 as therapeutic target [44]. The presence of cadherin and desmosomal complexes expand the realm of possibilities for modulating catenin signaling capacity in cancer cells, presenting novel therapeutic strategies for combating metastatic dissemination in colorectal cancer, and being particularly relevant for poorly-differentiated tumors.

### Supplementary Information

**Additional file 1.** Supplementary methodology.

**Additional file 2:** **Fig. S1.** Comparison among CDH17-immunoprecipitations in different colon cancer cell lines. **Fig. S2.** DSC1 regulates cell adhesion, migration and invasion. **Fig. S3.** DSC1 silencing inhibits phospho-JNK and Src expression. **Fig. S4.** DSC1 expression levels are associated with poor prognosis in the mesenchymal subtypes of colorectal cancer. **Fig. S5.** CDH17 presents a NLV motif in the extracellular domain.

**Additional file 3:** **Table S1.** List of CDH17 co-immunoprecipitated proteins in HT-29 and RKO cell lines. **Table S2.** CDH17 co-immunoprecipitated proteins in different colon cancer cell lines.

## Data Availability

The immunoprecipitation datasets generated during and/or analyzed during the current study are available from the corresponding author on reasonable request.
