# Supplementary material for: A complex of cadherin 17 with desmocollin 1 and p120-catenin regulates colorectal cancer migration and invasion according to the cell phenotype
Source: J Exp Clin Cancer Res. 2024 Jan 24;43:31. doi: 10.1186/s13046-024-02956-6 (PMC10807196; doi:10.1186/s13046-024-02956-6)
Supplement: Supplementary file 1 — Additional file 1. Supplementary methodology. [file 13046_2024_2956_MOESM1_ESM.docx]

**Additional information**

**Supplementary methodology**

**Cell transfection and silencing experiments**

The siRNAs against CDH17 (SASI_HS_00166354), DSC1 (SASI_HS01_00172856), p120-catenin isoform 1 (GGAUCACAGUCACCUUCUAUAdTdT) and control were purchased from Sigma-Aldrich. The esiRNA targeting DSC1 (EHU077291), used for in vivo assays, was from Sigma-Aldrich. Expression vectors pcDNA3.1 CDH17 wt, pcDNA3.1 CDH17 RAD and pcDNA3.1 CDH17 Fc were previously described ^1^. The vectors pcDNA3.1 CDH17 wt and pcDNA3.1 CDH17 Fc encoding for full length CDH17 and the CDH17 ectodomain fused to the immunoglobulin Fc-fragment, respectively, were transfected into RKO and HT-29 cells 48 h before lysis and CDH17 immunoprecipitation. The siRNAs and the vectors pcDNA3.1 CDH17 RAD were transfected in KM12SM, HT-29, SW620 and HCT-116 cells 24-48 h before in vitro assays. DSC1 esiRNA was transfected in KM12SM, HT-29, SW620 and HCT-116 cells 48 h before inoculation in mice. Vectors and siRNAs were transfected with JetPRime (Polyplus Transfection) according to manufacturer’s instructions.

**Confocal Microscopy**

KM12SM cells cultured to 70 % confluence on Matrigel-coated cover slides were fixed with 4% paraformaldehyde in PBS and permeabilized with 1% Triton X-100 in PBS. Then, cells were incubated with blocking medium (40 μg/mL human γ-globulin in PBS) for 1 h, followed by incubation with anti-CDH17, anti-DSC1 and anti-p120-catenin at 10 μg/mL for 1 h. After washing, cells were incubated with anti-rabbit IgG and anti-mouse IgG coupled with Alexa-fluor 488 or Alexa-fluor 647 (Invitrogen), respectively. Finally, fixed cells were washed and incubated with 4,6-diamidino-2-phenylindole (DAPI). Cover slides were mounted with Fluorescence Mounting Medium (Dako). Microscope images were obtained in a TCS-SP5-AOBS confocal microscope (Leica) using a 63x oil immersion objective.

**Western blot and subcellular fractionation**

For Western blot, cells were lysed with the same lysis buffer used for immunoprecipitation and protein extracts (50 μg) were resolved in SDS-PAGE and transferred to nitrocellulose membranes. Blots were incubated with primary antibodies for 16 h followed by incubation with HRP-conjugated secondary antibodies for 2 h (Thermo Fisher Scientific). Bands were visualized with SuperSignal West Pico Chemiluminescent Substrate (Thermo Fisher Scientific) in the Chemidoc Imaging System device (Bio-Rad) and quantified using MultiGauge software (Fujifilm).

Subcellular fractionation was carried out using the subcellular Protein Fractionation Kit for Cultured Cells (Thermo-Scientific). Total amount of protein in each fraction was assessed by Bradford assay, and a 5% of that protein content (ranging from 60 μg in the cytosolic fraction to 10 μg in the nuclear fraction) was resolved by SDS-PAGE and analyzed by Western blot.

**Mass spectrometry of immunoprecipitated proteins**

For proteomic analysis, 1 mg of cell lysates were immunoprecipitated as before, and the coimmunoprecipitated proteins were digested with trypsin. Peptides were loaded onto an AcclaimPepMap 100 precolumn (Thermo Fisher Scientific) and separated on an AcclaimPepMAp 100 C18 25 cm column (Thermo Fisher Scientific) using a flow rate of 300 nL/minute in a 100-minute gradient from 100 % Buffer A (0.1% formic acid, 2% acetonitrile in water) to 95 % Buffer B (0.1 % formic acid in acetonitrile) on a nanoEasy-nLC 1000 (Proxeon) coupled to a nanoelectrospay ion source (Thermo Fisher Scientific). Mass spectra were acquired in a Q-Exactive instrument (Thermo Fisher Scientific.Waltham. MA.USA). For ionization, 2000 V of liquid junction voltage and 270°C capillary temperature were used. The full scan method employed a *m/z* 400–1500 mass selection, an Orbitrap resolution of 70,000 (at m/z 200), a target automatic gain control (AGC) value of 3e6, and maximum injection times of 100 ms. After the survey scan, the 15 most intense precursor ions were selected for MS/MS fragmentation. Fragmentation was performed with a normalized collision energy of 27 eV and MS/MS scans were acquired with a starting mass of m/z 100, AGC target was 2e5, resolution of 17,500 (at m/z 200), intensity threshold of 8e3, isolation window of 2 *m/z* units. MS data were analysed with Proteome Discoverer v.1.4.1.14 (Thermo Fisher Scientific) using standardized workflows. A fold-change threshold of 3 compared to control antibody immunoprecipitated proteins was fixed for number of peptide spectral matches (PSM). Identified proteins whose functions imply unspecific protein-protein interaction (as chaperonins, ribosome proteins, proteasome proteins, etc.), as well as proteins involved in vesicle traffic to cell membrane were removed.

**In silico analysis of cancer patients**

To test DSC1 value in prognosis in different solid tumors, independent external cohorts of patients were used. Analyses were performed in the GSE39582 (with 585 colon cancer samples classified by CMS and CRIS subtypes. The values of mRNA expression levels were normalized by calculating z-scores, populations were divided in DSC1 high or low expression by the best cut-off method, and patient survivals were analyzed by log-rank test. In addition, we employed the web tools: GEPIA2 (<http://gepia2.cancer-pku.cn>) with transcriptomic data of lung cancer (n=353), kidney cancer (599) and melanoma (n=984), Kaplan-Meier Plotter (<https://kmplot.com/analysis/>), with mRNA data for lung adenocarcinoma (n=513) and kidney renal clear cell carcinoma (n=530), and OSSKCM (<https://bioinfo.henu.edu.cn/Melanoma/Melanoma_GSE22155_6102.jsp>) with 1085 samples of skin cutaneous melanoma.

1. Bartolomé RA, Peláez-García A, Gomez I, Torres S, Fernandez-Aceñero MJ, Escudero-Paniagua B*, et al.* An RGD motif present in cadherin 17 induces integrin activation and tumor growth. J Biol Chem 2014; 289**:** 34801-34814.
