# Supplementary material for: A complex of cadherin 17 with desmocollin 1 and p120-catenin regulates colorectal cancer migration and invasion according to the cell phenotype
Source: J Exp Clin Cancer Res. 2024 Jan 24;43:31. doi: 10.1186/s13046-024-02956-6 (PMC10807196; doi:10.1186/s13046-024-02956-6)
Supplement: Supplementary file 2 — Additional file 2: Fig. S1. Comparison among CDH17-immunoprecipitations in different colon cancer cell lines. Fig. S2. DSC1 regulates cell adhesion, migration and invasion. Fig. S3. DSC1 silencing inhibits phospho-JNK and Src expression. Fig. S4. DSC1 expression levels are associated with poor prognosis in the mesenchymal subtypes of colorectal cancer. Fig. S5. CDH17 presents a NLV motif in the extracellular domain. [file 13046_2024_2956_MOESM2_ESM.pdf]

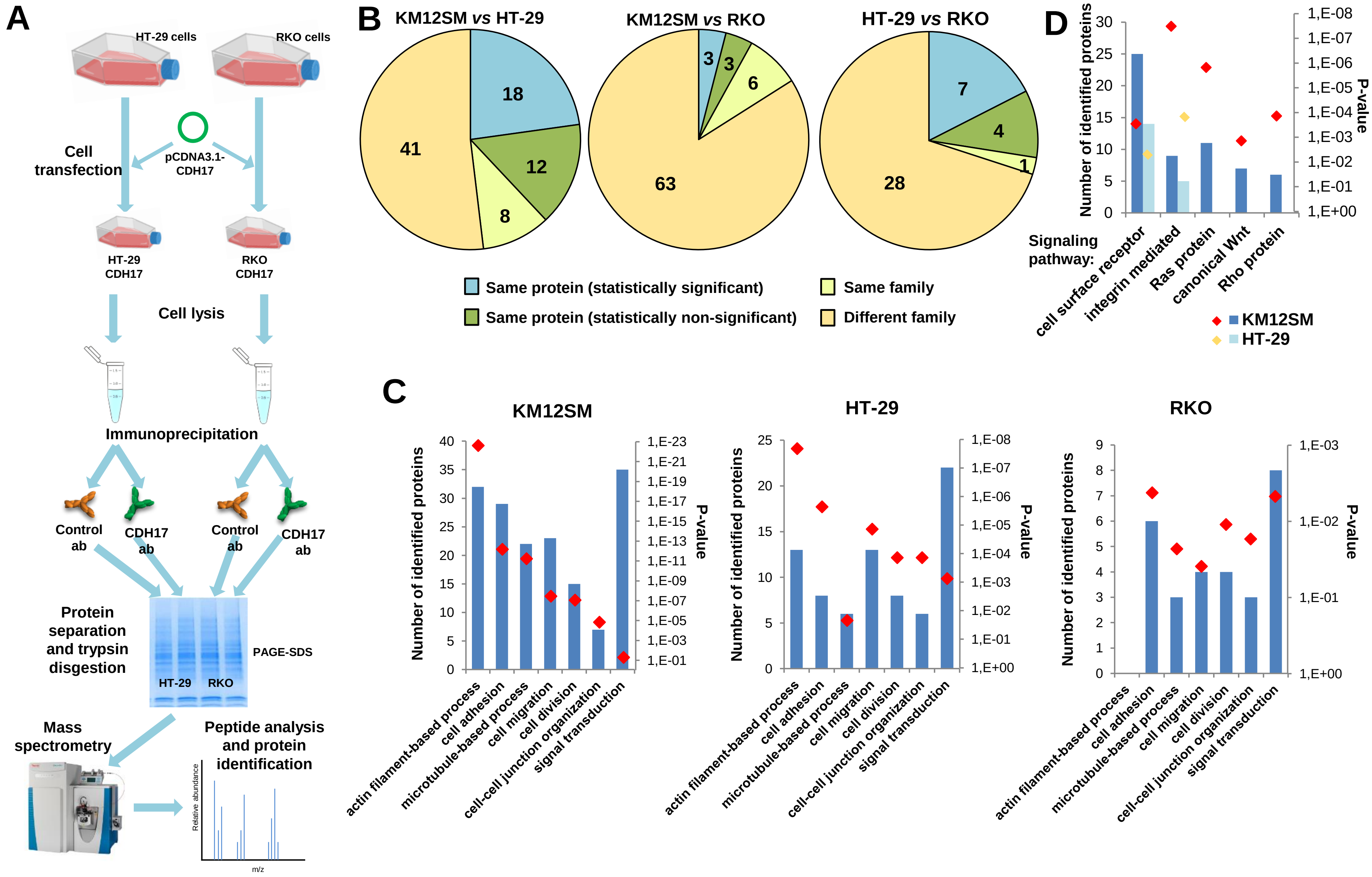

Figure S1. Comparison among CDH17-immunoprecipitations in different colon cancer cell lines. (A) Schematic representation of the immunoprecipitation of CDH17 in transfected cells and mass spectrometry analysis of the immunoprecipitates. (B) CDH17-co-immunoprecipitated proteins in KM12SM were compared with the mass spectrometry identified-proteins in CDH17-specific immunoprecipitates of HT-29 and RKO cells transfected with vectors encoding for CDH17. Also, identified proteins in HT-29 and RKO cells were compared. The number of identifies proteins in each condition is indicated. (C) Major biological functions identified in CDH17 immunoprecipitates according to Gene Ontology. Histograms indicate number of identified proteins; ◊, p-value. (D) Signaling pathways identified in CDH17 immunoprecipitates in KM12SM and HT-29 cells. No signaling pathway was identified in RKO cells.

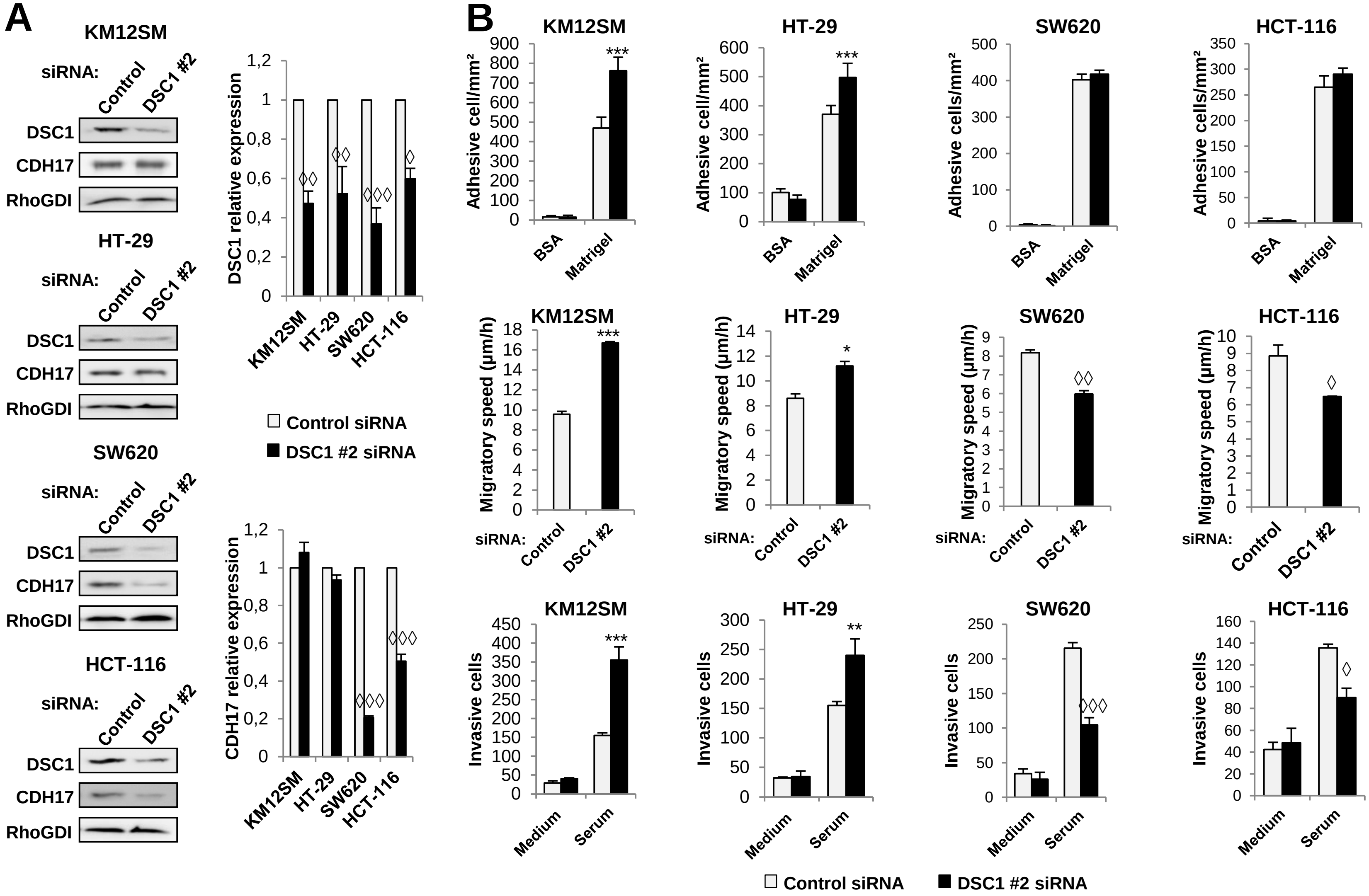

Figure S2. DSC1 regulates cell adhesion, migration and invasion. (A) The indicated colon cancer cell lines were transfected with a second siRNA targeting DSC1 or a scramble siRNA (control). After 48h, cells were lysed and the extracts analyzed by Western blot to verify the silencing. RhoGDI was used as loading control. Quantifications of DSC1 and CDH17 expression levels from 3 independent experiments are shown. The transfection of DSC1 #2 siRNA caused a significant reduction in the expression levels of DSC1 and/or CDH17 (◊, $p < 0.05$; ◊◊, $p < 0.01$; ◊◊◊, $p < 0.001$). (B) The same transfectants were subjected to cell adhesion, wound healing and invasion assays. Cell adhesion, migration or invasion was significantly increased (*, $p < 0.05$; **, $p < 0.01$; ***, $p < 0.001$) or decreased (◊, $p < 0.05$; ◊◊, $p < 0.01$; ◊◊◊, $p < 0.001$) by DSC1 silencing. Data of a representative experiment out of three are shown as average ± standard deviation.

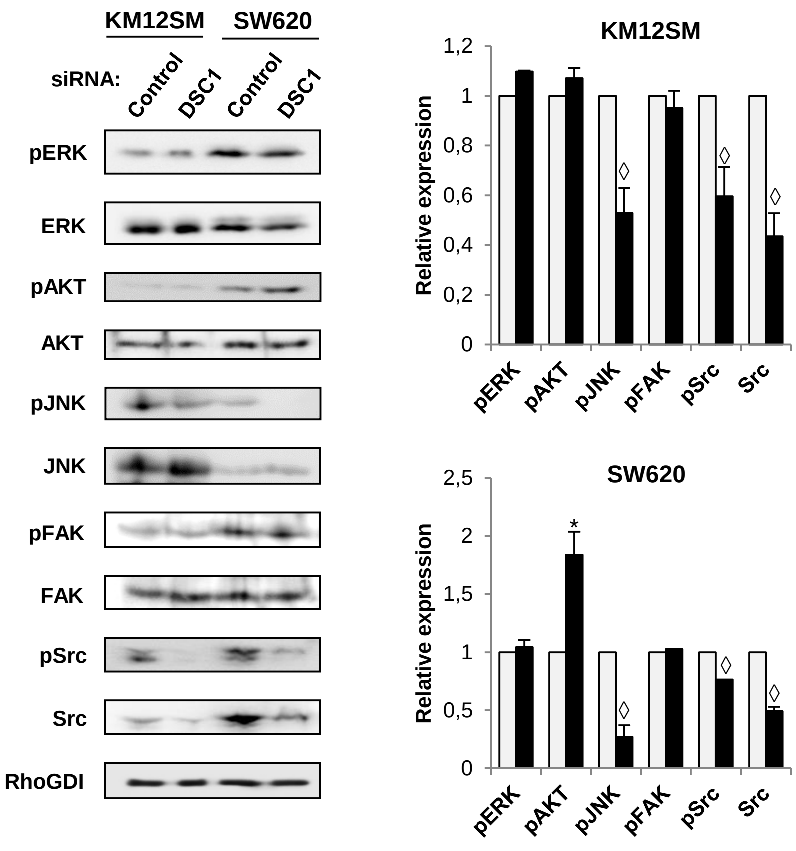

Figure S3. DSC1 silencing inhibits phospho-JNK and Src expression. The indicated colon cancer cell lines were transfected with control or DSC1-targeting siRNAs, lysed and the extracts were analyzed by Western blot to detect the phosphorylated forms of the indicated signaling proteins, and their corresponding total proteins. Quantification of phosphoproteins are relative to their total counterparts, whereas quantification of total Src was relative to RhoGDI. Quantification of the indicated (phospho-)protein expression levels from 3 independent experiments is shown as average ± standard deviation. DSC1 silencing caused a significant increment (*, $p < 0.05$) or decrement (◊, $p < 0.05$) in the expression of the indicated (phospho-)protein.

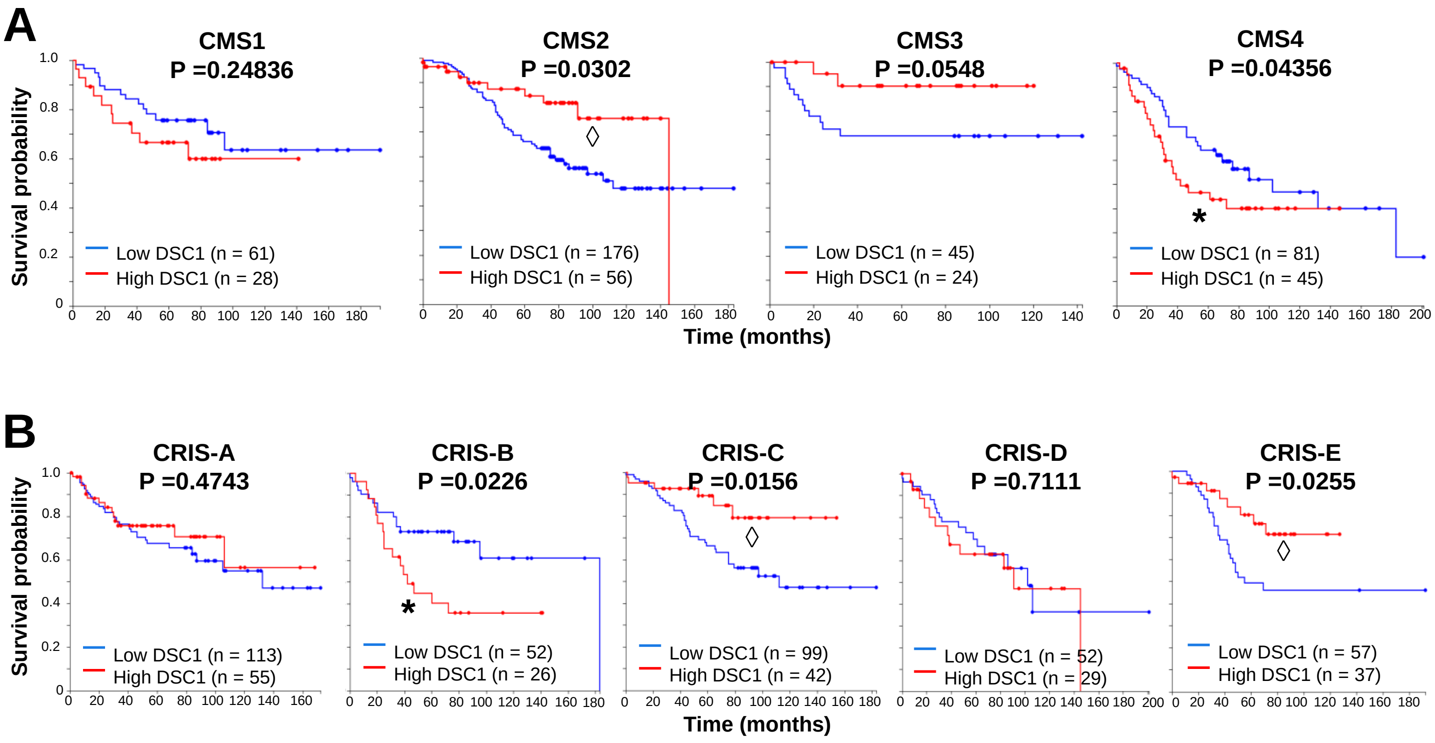

Figure S4. DSC1 expression levels are associated with poor prognosis in the mesenchymal subtypes of colorectal cancer. DSC1 mRNA expression levels were significantly associated with poor prognosis (*, $p < 0.05$) or good prognosis (◊, $p < 0.05$) in the indicated subtypes (Consensus Molecular Subtypes; Colorectal Intrinsic Subtypes) of colorectal cancer.

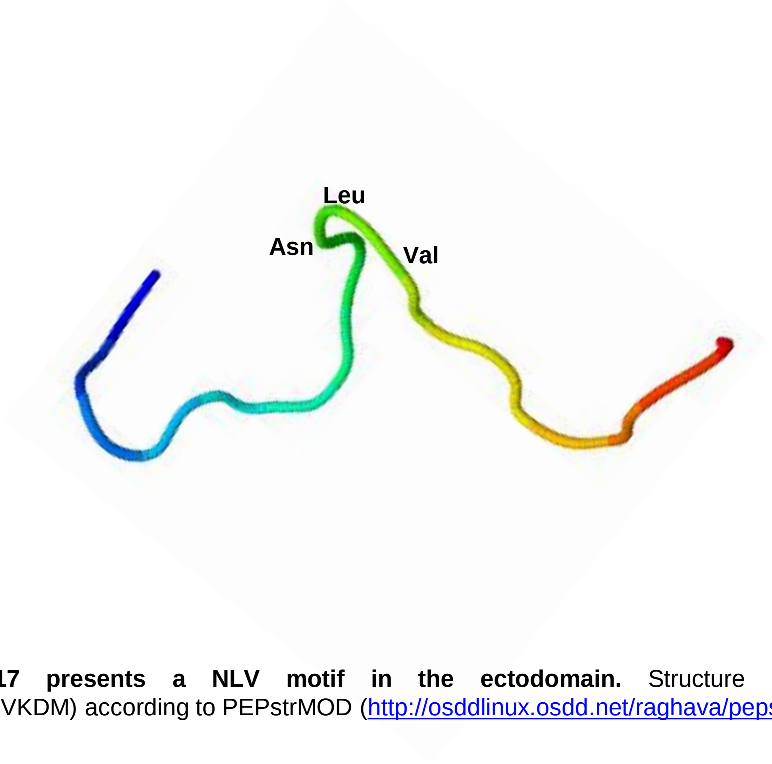

Figure S5. CDH17 presents a NLV motif in the ectodomain. Structure of the CDH17-NLV peptide (LNPAKNPSYNLVISVKDM) according to PEPstrMOD (http://osddlinux.osdd.net/raghava/pepstrmod/index.php).
