# Supplementary material for: A complex of cadherin 17 with desmocollin 1 and p120-catenin regulates colorectal cancer migration and invasion according to the cell phenotype
Source: J Exp Clin Cancer Res. 2024 Jan 24;43:31. doi: 10.1186/s13046-024-02956-6 (PMC10807196; doi:10.1186/s13046-024-02956-6)
Supplement: Supplementary file 3 — Additional file 3: Table S1. List of CDH17 co-immunoprecipitated proteins in HT-29 and RKO cell lines. Table S2. CDH17 co-immunoprecipitated proteins in different colon cancer cell lines. [file 13046_2024_2956_MOESM3_ESM.pdf]

Table S1: List of CDH17 immunoprecipitated proteins in HT-29 and RKO cell lines.

**Identified proteins in HT-29 cell line**			Control IP			CDH17 IP		
Accession	Name	Description	Score	# Peptides	# PSM	Score	# Peptides	# PSM
Q9BQE3	TUBA1C	Tubulin alpha-1C chain	126,89	4	4	728,73	10	23
P14923	JUP	Junction plakoglobin	44,52	1	1	513,25	13	16
Q08554	DSC1	Desmocollin-1	35,68	1	1	108,28	3	4
P07437	TUBB5	Tubulin beta chain 5	146,11	3	3	760,88	9	23
P68366	TUBA4A	Tubulin alpha-4A chain	126,89	4	4	477,24	8	18
P68371	TUBB2C	Tubulin beta-2C chain	125,88	3	3	651,02	8	19
P28799	GRN	Granulins	38,68	1	1	239,18	7	12
P35579	MYH9	Myosin-9				591,42	17	19
P00352	ALDH1A1	Retinal dehydrogenase 1				390,84	11	11
P12814	ACTN1	Alpha-actinin-1				337,92	10	10
P29508	SERPINB3	Serpin B3				30,87	1	1
P04040	CAT	Catalase				149,88	4	4
P02452	COL1A1	Collagen alpha-1(I) chain				166,31	5	6
P08123	COL1A2	Collagen alpha-2(I) chain				99,35	3	3
P31151	S100A7	Protein S100-A7				99,02	2	3
P15311	EZR	Ezrin				213,24	8	8
P07737	PFN1	Profilin-1	48,38	1	1	167,08	3	4
Q96AG4	LRRC59	Leucine-rich repeat-containing protein 59				85,91	3	3
P05089	ARG1	Arginase-1				64,30	2	2
Q13813	SPTAN1	Spectrin alpha chain, brain				29,45	1	1
O60701	UGDH	UDP-glucose 6-dehydrogenase				119,34	5	5
P30740	SERPINB1	Leukocyte elastase inhibitor				176,49	5	5
Q00535	CDK5	Cell division protein kinase 5				39,61	2	2
O60610	DIAPH1	Protein diaphanous homolog 1				40,19	1	2
O75369	FLNB	Filamin-B				318,71	11	11
P46940	IQGAP1	Ras GTPase-activating-like protein IQGAP1				106,34	5	5
O75874	IDH1	Isocitrate dehydrogenase [$NADP^+$] 1				85,13	3	3
P36873	PPP1CC	Protein phosphatase PP1-gamma catalytic subunit				132,39	2	3
Q9Y490	TLN1	Talin-1				80,34	2	2
Q16181	SEPT7	Septin-7				73,23	2	2
P07384	CAPN1	Calpain-1 catalytic subunit				71,85	2	2
Q12864	CDH17	Cadherin-17				60,72	2	2
P53041	PPP5C	Protein phosphatase 5 catalytic subunit				59,59	2	2
Q9P258	RCC2	Protein RCC2				55,03	2	2
Q9NVA2	SEPT11	Septin-11				51,33	2	2
O15144	ARPC2	Actin-related protein 2/3 complex subunit 2				50,91	2	2
P17931	LGALS3	Galectin-3				46,55	2	2
P30085	CMPK1	UMP-CMP kinase				44,13	2	2
P20742	PZP	Pregnancy zone protein				41,74	2	2
P60953	CDC42	Cell division control protein 42 homolog				43,68	1	2

Identified proteins in RKO cell line			Control IP			CDH17 IP		
Accession	Name	Description	Score	# Peptides	# PSM	Score	# Peptides	# PSM
P14923	JUP	Junction plakoglobin	64,35	2	3	184,26	4	6
P29508	SERPINB3	Serpin B3	28,49	1	1	94,49	3	4
Q08554	DSC1	Desmocollin-1	67,49	1	1	131,75	3	4
Q27J81	INF2	Inverted formin-2				252,85	6	7
P12273	PIP	Prolactin-inducible protein	62,60	2	2	30,79	1	1
P05089	ARG1	Arginase-1				61,62	2	2
P04040	CAT	Catalase				111,93	4	4
Q96AG4	LRRC59	Leucine-rich repeat-containing protein 59	37,54	1	1	62,04	3	3
Q9Y315	DERA	Deoxyribose-phosphate aldolase	57,98	1	1	46,44	1	1
O00139	KIF2A	Kinesin-like protein KIF2A				66,08	2	2
P04196	HRG	Histidine-rich glycoprotein				74,00	1	2
Q9BW19	KIFC1	Kinesin-like protein KIFC1				70,01	2	2
Q12864	CDH17	Cadherin-17				55,16	2	2
P09382	LGALS1	Galectin-1				23,20	1	1
P63172	DYNLT1	Dynein light chain Tctex-type 1				30,46	1	1

Table S2: CDH17 co-immunoprecipitated proteins in different colon cancer cell lines.

Proteins identified in KM12SM cells			RKO	HT-29
**Accesion**	**Protein name**	**Gene name**	**Gene name**	**Gene name**
Q12864	Cadherin-17	CDH17	CDH17	CDH17
P14923	Junction plakoglobin	JUP	JUP	JUP
P35221	Catenin alpha-1	CTNNA1		
P16422	Epithelial cell adhesion molecule	EPCAM		EPCAM
O43813	LanC-like protein 1	LANCL1		
P23528	Cofilin-1	CFL1		CFL1
P16070	CD44 antigen	CD44		
Q8WX93	Isoform 4 of Palladin	PALLD		
P52565	Rho GDP-dissociation inhibitor 1	ARHGDIA		
Q12792	Twinfilin-1	TWF1		
P17931	Galectin-3	LGALS3	LGALS1	LGALS3
P37802	Transgelin-2	TAGLN2		TAGLN2
Q9NRL3	Striatin-4	STRN4		STRN4
Q86W34	Archaemetzincin-2	AMZ2		
O75369	Filamin-B	FLNB		FLNB
Q13813	Spectrin alpha chain, brain	SPTAN1		SPTAN1
P68371	Tubulin beta-4B chain	TUBB4B		TUBB2C
Q9BQE3	Tubulin alpha-1C chain	TUBA1C		TUBA1C
Q9Y490	Talin-1	TLN1		TLN1
P12814	Alpha-actinin-1	ACTN1	ACTN1	ACTN1
P68366	Tubulin alpha-4A chain	TUBA4A		TUBA4A
P16144	Integrin beta-4	ITGB4		
Q9BUF5	Tubulin beta-6 chain	TUBB6		TUBB5
P33176	Kinesin-like protein KIF5A	KIF5B	KIF2A	KIF5B
Q14204	Cytoplasmic dynein 1 heavy chain 1	DYNC1H1	DYNLT1	DYNC1H1
O60610	Protein diaphanous homolog 1	DIAPH1		DIAPH1
P15311	Ezrin	EZR		EZR
Q13576	Ras GTPase-activating-like protein IQGAP2	IQGAP2		IQGAP1
Q7Z406	Myosin-14	MYH14		MYH9
P18206	Vinculin	VCL		
P27816	Microtubule-associated protein 4	MAP4		
P29966	Myristoylated alanine-rich C-kinase substrate	MARCKS		
P52732	Kinesin-like protein KIF11	KIF11	KIFC1	
Q9HC35	Echinoderm microtubule-associated protein-like 4	EML4		
P35442	Thrombospondin-2	THBS2		
P06493	Cyclin-dependent kinase 1	CDK1		CDK5
Q15019	Septin-2	SEPT2		SEPT2
O60716	Catenin delta-1	CTNND1		CTNND1
P62136	Protein phosphatase PP1-alpha catalytic subunit	PPP1CA		PPP1CC
P30622	CAP-Gly domain-containing linker protein 1	CLIP1		
P05556	Integrin beta-1	ITGB1		
Q12965	Unconventional myosin-Ie	MYO1E		
Q13045	Protein flightless-1 homolog	FLII		
P60953	Cell division control protein 42 homolog	CDC42		CDC42
Q9Y295	Developmentally-regulated GTP-binding protein 1	DRG1		
P61160	Actin-related protein 2	ACTR2		

Proteins identified in KM12SM cells			RKO	HT-29
**Accesion**	**Protein name**	**Gene name**	**Gene name**	**Gene name**
Q16181	Septin-7	SEPT7	SEPT7	SEPT7
Q7L576	Cytoplasmic FMR1-interacting protein 1	CYFIP1		
P07737	Profilin-1	PFN1	PFN1	PFN1
P49006	MARCKS-related protein	MARCKSL1		
O14974	Protein phosphatase 1 regulatory subunit 12A	PPP1R12A		
P35222	Catenin beta-1	CTNNB1		
P23229	Integrin alpha-6	ITGA6		
Q9UHD8	Isoform 7 of Septin-9	SEPT9		SEPT9
P47756	F-actin-capping protein subunit beta	CAPZB		CAPZB
Q9Y2A7	Nck-associated protein 1	NCKAP1		
P20936	Ras GTPase-activating protein 1	RASA1		
O15144	Actin-related protein 2/3 complex subunit 2	ARPC2		ARPC2
Q27J81	Inverted formin-2	INF2	INF2	INF2
P18827	Syndecan-1	SDC1		
P17655	Calpain-2 catalytic subunit	CAPN2		CAPN1
Q9NVA2	Septin-11	SEPT11		SEPT11
Q01970	Phosphoinositide phospholipase C beta3	PLCB3		
P50570	Dynamin-2	DNM2		
Q9P258	Protein RCC2	RCC2		RCC2
Q92974	Rho guanine nucleotide exchange factor 2	ARHGEF2		ARHGEF2
Q07866	Kinesin light chain 1	KLC1		
Q09666	Neuroblast differentiation-associated protein AHNAK	AHNAK		
Q9P035	3-hydroxyacyl-CoA dehydratase 3	PTPLAD1		
O75116	Rho-associated protein kinase 2	ROCK2		
Q5H9R7	protein phosphatase 6 regulatory subunit 3	PPP6R3		
P30740	Leukocyte elastase inhibitor	SERPINB1	SERPINB3	SERPINB1
Q9BQL6	Fermitin family homolog 1	FERMT1		
O94804	Serine/threonine-protein kinase 10	STK10		
Q9H3S7	Tyrosine-protein phosphatase non-receptor type 23	PTPN23		
Q86TR8	Serine/threonine-protein kinase MRCK beta	CDC42BPB		
Q9Y3S1	Serine/threonine-protein kinase WNK2	WNK2		
O00743	protein phosphatase 6 catalytic subunit	PPP6C		
Q5VZK9	Leucine-rich repeat-containing protein 16A	LRRC16A	LRRC59	LRRC59

Legend (cell colours in the RKO and HT-29 columns):
- Blue: Same protein (statistically significant)
- Green: Same protein (non-statistically significant)
- Yellow: Protein from the same family

Blue: SEPT7 (HT-29), PFN1 (HT-29), ARPC2 (HT-29), INF2 (RKO), SEPT11 (HT-29), RCC2 (HT-29), SERPINB1 (HT-29).
Green: SEPT7 (RKO), PFN1 (RKO), SEPT9 (HT-29), CAPZB (HT-29), INF2 (HT-29), ARHGEF2 (HT-29).
Yellow: CAPN1 (HT-29), SERPINB3 (RKO), LRRC59 (RKO), LRRC59 (HT-29).
